# The Extent and Nature of Lived Experience Engagement in the Development of Australian Clinical Practice Guidelines, 2014–2025: A Scoping Review

**DOI:** 10.5694/mja2.70132

**Published:** 2026-02-02

**Authors:** Anneliese Synnot, Naomi MacPherson, Thomas Benning, Bernard Tso, Chuyue Wang, Antonia Arfaras, Brian A. Beh, Vanessa Cullen, Jessica D'Lima, Tony Finneran, David C. Fry, Michelle King, Alexander Meredith, Adrian O'Malley, Joanne Muller, Tari Turner, Samantha P. Chakraborty

**Affiliations:** ^1^ School of Public Health and Preventive Medicine Monash University Melbourne Victoria Australia; ^2^ Australian Living Evidence Collaboration Lived Experience Advisory Group Monash University Melbourne Victoria Australia; ^3^ Queensland Aphasia Research Centre University of Queensland Brisbane Queensland Australia; ^4^ Surgical Treatment and Rehabilitation Service (STARS) University of Queensland and Metro North Health Brisbane Queensland Australia; ^5^ Cochrane Australia Monash University Melbourne Victoria Australia

**Keywords:** Australia, guidelines as topic, lived experience engagement

## Abstract

**Objectives:**

To examine the extent and nature of lived experience engagement in Australian clinical practice guideline development.

**Study Design:**

Scoping review of Australian clinical practice guidelines published 1 January 2014–20 March 2025 that reported using a systematic search method and standardised methods for appraising evidence quality and certainty.

**Data Sources:**

PubMed, Guidelines International Network library, Google Scholar, the websites of all 25 Australian medical colleges, the Cancer Council, the Heart Foundation, the Stroke Foundation, the National Blood Authority and Caring for Australians and New Zealanders with Kidney Impairment.

**Data Synthesis:**

One hundred and fifty guidelines met the inclusion criteria; 108 (72%) reported some degree of lived experience engagement in their development, of which 98 (91%) described engagement through all development stages and 95 (88%) reported their inclusion as guideline panel members. Other methods of engagement included participation in lived experience panels and advisory groups (10 guidelines, 9%) and online surveys (5 guidelines, 5%). Ninety‐seven of 108 guidelines (90%) with lived experience engagement reported that people with lived experience were asked to decide, advise or vote on recommendations or guideline content. One person with lived experience participated in the development process for 61 guidelines (56%), two people for 14 guidelines (13%), 3–10 people for 19 guidelines (18%) and more than 10 people for 10 guidelines (9%). Little information was reported about the characteristics of participating people with lived experience. Sixty guidelines (56%) reported remunerating people with lived experience for their participation, 49 guidelines (45%) reported that they received practical support and 41 guidelines (38%) reported that group dynamics were managed to support lived experience engagement.

**Conclusions:**

It is encouraging that most Australian guidelines published during 2014–2025 reported at least some lived experience engagement in their development. However, extensive lived experience engagement was not reported for the vast majority of guidelines. The engagement of people with lived experience in guideline development needs to be improved to ensure that their values, views and preferences are reflected.

## Plain Language Summary

**The known**: Involving people with lived experience of health conditions when developing clinical practice guidelines in Australia is required by the National Health and Medical Research Council.

**The new**: Most Australian guidelines published during 2014–2025 included people with lived experience throughout their development, but the degree of engagement was typically quite limited.

**The implications**: Increasing lived experience engagement in guideline development will better ensure that their values and preferences are appropriately considered.

## 1 Introduction

Clinical practice guidelines are recommendations for clinicians making health care decisions. Major guideline bodies, such as the World Health Organization, the United States Institute of Medicine and the National Health and Medical Research Council (NHMRC), recommend that the guideline development process include people with lived experience of the topic or condition covered by the guidelines, members of their families or their representatives [1, 2, 3]. Their engagement can take many forms, such as membership of guideline development groups and participation in prioritisation surveys, workshops and interviews [4, 5, 6], at any stage of the guideline development process [7], leading to more person‐centred guidelines [8, 9].

The extent of and expectations regarding lived experience engagement in health research have shifted considerably since the early 2000s [10, 11, 12]. Areas that have attracted greater attention include sharing power with people with lived experience [13], their meaningful and effective engagement [14], and increasing equity, in part by reducing barriers to participation for people from groups who are less heard [15]. Nevertheless, shifts in the guideline development community have been slow; recent studies reported that only 8% of United States guideline development organisations (2017) [16] and 11% of Latin American guidelines (2022) [17] included people with lived experience of the topic in their guideline development groups.

In Australia, guideline development is decentralised, undertaken by government health departments, medical colleges, disease‐specific charity groups and research institutions [18]. The NHMRC produces a small number of guidelines and has a formal approval process for externally produced guidelines developed using NHMRC methods [3]. According to NHMRC standards, guideline developers must involve people with lived experience in guideline development groups and throughout the development process, and report how they were recruited, engaged and supported [3, 19]. A 2014 analysis of Australian guidelines, undertaken prior to the publication of the first (2016) NHMRC guideline standards, found that 14% reported lived experience engagement in their development [18]. Lived experience engagement in Australian guideline development has not since been investigated.

We therefore examined the extent and nature of lived experience engagement in Australian clinical practice guideline development.

## 2 Methods

We followed JBI guidance for the conduct of scoping reviews [20, 21] and report our scoping review according to the Preferred Reporting Items for Systematic Reviews and Meta‐Analysis extension for Scoping Reviews (PRISMA‐ScR) [22]. We did not publish the review protocol.

### 2.1 Participants

We defined ‘people with lived experience’ as people with lived experience of health conditions, patients and potential patients, informal caregivers, people who use health care services, and community members and their representatives, including organisational representatives.

### 2.2 Core Concepts

We defined ‘lived experience engagement’ as active involvement in a bi‐directional relationship that results in informed decision‐making at any stage of the guideline development process [23]. We used an operational definition of the engagement of one or more people with lived experience at any stage of the process, with the exception of the public consultation stage and formal searches for information about patients' preferences, values and experiences to inform recommendation development. We defined the extent of lived experience engagement as its prevalence, and its nature as its key features, most frequently recruitment, guideline stages during which their engagement was reported, methods of engagement and training and support provided.

### 2.3 Context

We included Australian guidelines published during 1 January 2014–20 March 2025. This period was selected because of the growth in lived experience engagement expectations, practice and methods during this period, and our desire to examine current practice. We used the US Institute of Medicine definition of guidelines: ‘statements that include recommendations, intended to optimise patient care, that are informed by a systematic review of evidence and an assessment of the benefits and harms of alternative care options’ [2]. We included guidelines that clearly described a systematic search (e.g., questions framed using population, intervention, comparison and outcomes, databases or search strings) and methods for appraising the quality and certainty of the evidence (e.g., Grading of Recommendations Assessment, Development, and Evaluation [GRADE] [24]). We included guidelines designed to guide clinical practice related to any population group or health condition, apart from those pertaining to public health and allied health. We selected guidelines with a national scope, including those that applied to both Australia and New Zealand.

### 2.4 Document Sources

We included guideline documents and any related reports, such as technical reports or journal articles, that described guideline development methods.

### 2.5 Search Strategy

With the assistance of an information specialist, we searched the following databases and document repositories on 20 March 2025:

- PubMed (search string: Australia*[ti] AND (guideline*[ti] OR guideline[pt]));
- Guidelines International Network Library (https://guidelines.ebmportal.com) (filter: Australia).

We also searched the following databases and sources: ECRI Guidelines Trust (https://guidelines.ecri.org); MAGICapp (https://app.magicapp.org); Google ‘Australian guideline’ and Google Scholar ‘Australian guideline’ (first five result pages each checked); and the websites of all 25 Australian medical colleges, the Cancer Council (https://www.cancer.org.au), the Heart Foundation (https://www.heartfoundation.org.au), the Stroke Foundation (https://strokefoundation.org.au), the National Blood Authority (https://www.blood.gov.au) and Caring for Australians and New Zealanders with Kidney Impairment (https://www.cariguidelines.org). Search results were collated and duplicates removed in EndNote 20; the documents were screened in Covidence.

### 2.6 Selection Process

Two reviewers (from authors NM, TB and CW) independently screened the titles and abstracts of items identified by the searches; disagreements were resolved by consensus or discussion with a third reviewer (one of the authors AS and SC). One reviewer (from NM, TB and CW) independently screened the full text of documents selected by screening; a second reviewer (SC) checked about 10% of these documents, and merged any guideline chapters as single guideline documents, as appropriate.

### 2.7 Data Charting Items and Process

Two authors (AS and TT) devised and piloted a standardised template in Excel (Microsoft) for data charting. We drew upon existing frameworks [25, 26] to inform how we categorised data items (template: Table S1). Briefly, we recorded the guideline topic, year of publication, whether the guideline met any of the NHMRC standards related to people with lived experience of the topic, and whether the guideline was developed with any lived experience engagement. We used the 2022 NHMRC standards [27] for data charting (the 2025 standards [3] had not yet been published). For guidelines that reported lived experience engagement, we extracted information about their approach, such as the number and characteristics of the people engaged, the guideline stages in which they were involved, the methods of engagement and support provided to people with lived experience and guideline developers. We used PROGRESS‐Plus health equity characteristics to chart the diversity characteristics of the people with lived experience who were engaged in the guidelines [28, 29]. One reviewer (from TB, NM, BT, CW and AS) conducted data charting; queries were discussed with a senior author (AS or SC). A second reviewer (from AS, SC, BT and CW) checked all data charting, looked for errors and inconsistencies and directly amended the data charting spreadsheet.

### 2.8 Synthesis

To determine the extent of lived experience engagement in Australian guideline development, we calculated the proportion of guidelines in which one or more people with lived experience of the topic had been involved in their development. To determine the nature of lived experience engagement in Australian guidelines, we considered the key features of their engagement. We provide detailed accounts of a selection of guidelines with more extensive lived experience engagement.

### 2.9 Lived Experience Engagement in Our Scoping Review

We presented an early version of our review findings to an online meeting of the Australian Living Evidence Collaboration (https://livingevidence.org.au) 10‐member lived experience advisory group in September 2024. Feedback from the group led to the addition of further data charting items, refined how the results were presented, identified key messages and highlighted points that were included in the discussion. Group members were subsequently invited to comment on the manuscript and discuss changes with the authors at an online meeting.

## 3 Results

We identified 1367 potentially relevant records related to 1278 documents (some guidelines were reported in several publications or were published as chapters that we merged into single documents) in the searched databases, repositories and other sources. After removing 103 duplicates, we screened the titles and abstracts of 1175 unique items; we subsequently reviewed the full text of 485 documents deemed potentially relevant after screening titles and abstracts. After excluding 335 documents deemed to be ineligible for our review, we included 150 guidelines in our scoping review (Figure S1, Table S2).

### 3.1 Characteristics of Included Guidelines

The most frequent guideline topics were pregnancy, childbirth or the puerperium (41 guidelines), neoplasms (18 guidelines), diseases of the genitourinary system (17 guidelines), mental, behavioural or neurodevelopmental disorders (14 guidelines) and factors influencing health status or contact with health services (14 guidelines). A total of 108 guidelines (72%) described lived experience engagement in their development, and 42 guidelines (28%) did not; 32 guidelines had received NHMRC approval (Table 1).

**TABLE 1:** Characteristics of 150 Australian clinical practice guidelines that described a systematic search and methods for appraising the quality and certainty of the evidence, and published during 1 January 2014–20 March 2025.

Characteristic	Number
All guidelines	150
Guideline topic (International Classification of Diseases, eleventh revision category) [30]	
01 Certain infectious or parasitic diseases	4 (3%)
02 Neoplasms	18 (12%)
03 Diseases of the blood or blood‐forming organs	1 (1%)
04 Diseases of the immune system	1 (1%)
05 Endocrine, nutritional or metabolic diseases	3 (2%)
06 Mental, behavioural or neurodevelopmental disorders	14 (9%)
07 Sleep–wake disorders	0
08 Diseases of the nervous system	3 (2%)
09 Diseases of the visual system	0
10 Diseases of the ear or mastoid process	0
11 Diseases of the circulatory system	6 (4%)
12 Diseases of the respiratory system	7 (5%)
13 Diseases of the digestive system	1 (1%)
14 Diseases of the skin	0
15 Diseases of the musculoskeletal system or connective tissue	5 (3%)
16 Diseases of the genitourinary system	17 (11%)
17 Conditions related to sexual health	1 (1%)
18 Pregnancy, childbirth or the puerperium	41 (27%)
19 Certain conditions originating in the perinatal period	8 (5%)
20 Developmental anomalies	1 (1%)
21 Symptoms, signs or clinical findings, not elsewhere classified	1 (1%)
22 Injury, poisoning or certain other consequences of external causes	1 (1%)
23 External causes of morbidity or mortality	3 (2%)
24 Factors influencing health status or contact with health services	14 (9%)
Lived experience engagement	
Yes	108 (72%)
No	42 (28%)
Received National Health and Medical Research Council approval	
Yes	32 (21%)
No	118 (79%)

Ninety‐five guidelines (63%) reported that people with lived experience were included in the guideline development group, consistent with the NHMRC guideline development standards [27]; recruitment processes were described in 93 guidelines (62%), involvement processes in 104 (69%) and support processes (e.g., remuneration and practical support) in 76 (51%). Twenty‐two guidelines (15%) reported searches for information about patient preferences and values; 52 (35%) reported sending the guideline to patient organisations during public consultations (Table 2).

**TABLE 2:** Assessment of 150 Australian clinical practice guidelines published during 1 January 2014–20 March 2025 according to National Health and Medical Research Council standards related to lived experience engagement [27].

National Health and Medical Research Council standard	Number
All guidelines	150
People with lived experience participated in guideline development (mandatory)	108 (72%)
Guideline development group included people with lived experience (mandatory)	95 (63%)
Processes to recruit, involve and support people with lived experience described (mandatory)	
Recruit	93 (62%)
Involve	104 (69%)
Support	76 (51%)
Searched for evidence of patient preferences and values (desirable)	22 (15%)
Guideline sent to lived experience organisations during public consultation (mandatory)	52 (35%)

### 3.2 Nature of Lived Experience Engagement in Guidelines

Of the 108 guidelines that reported lived experience engagement, 81 reported using closed recruitment approaches (seeking people from existing groups, 70 [65%]; by personal invitation, 11 [10%]). People were recruited from lived experience groups for 82 guidelines (76%) or were known contacts of the guideline developers for 10 (9%) (Table 3).

**TABLE 3:** Nature of lived experience engagement in guideline development for 108 Australian clinical practice guidelines published during 1 January 2014–20 March 2025: Recruitment and characteristics.

Characteristic	Number
Guidelines that reported lived experience engagement	108
Recruitment approach^a^ [25]	
Open: fixed	11 (10%)
Open: flexible	2 (2%)
Closed: invitation	11 (10%)
Closed: existing group	70 (65%)
Closed: purposive sampling	0
Not reported	23 (21%)
Recruitment source [6]	
Lived experience groups	82 (76%)
Patient records from health care providers	1 (1%)
People with lived experience	0
Contacts of researcher or guideline developer	10 (9%)
Not reported	15 (14%)
Number of people with lived experience	
1	61 (56%)
2	14 (13%)
3–10	19 (18%)
More than 10	10 (9%)
Not reported	4 (4%)
Type of people with lived experience^a^	
Person with lived experience/patient	30 (28%)
Family member	16 (15%)
Advocate	7 (6%)
Organisational representative	14 (13%)
Not reported	69 (64%)
PROGRESS‐Plus characteristics [28, 29]	
Place of residence	5 (5%)
Race/culture/ethnicity/language	11 (10%)
Occupation	1 (1%)
Gender/sex^b^	1 (1%)
Religion	0
Education (limited)	1 (1%)
Socio‐economic status	1 (1%)
Social capital	0
Age	2 (2%)
Sexual orientation	0
Disability	2 (2%)
Not reported	94 (87%)

^a^ Multiple responses possible.

^b^ Sex refers to biological differences between males and females and gender refers to social roles and other traits generally associated with the sexes [29].

One person with lived experience was reported to have participated in the development process for 61 guidelines (56%), two people for 14 guidelines (13%), 3–10 people for 19 guidelines (18%) and more than 10 people for 10 guidelines (9%). The type of lived experience was not reported by 69 guidelines (64%); in 30 cases, they were people with direct lived experience (28%), in 16 cases family members (15%), in 14 cases organisational representatives (13%) and in 7 cases patient advocates (6%). Ninety‐four guidelines (88%) did not report the personal characteristics of people with lived experience; the characteristics most frequently reported were race/culture/ethnicity/language (11 guidelines, 10%) and place of residence (5 guidelines, 5%) (Table 3).

Eleven guidelines (10%) reported co‐designing lived experience engagement with people with lived experience. Ninety‐eight (91%) reported involving them throughout guideline development; when engagement was limited to specific stages, it was most frequently priority setting and topic selection (7 guidelines, 6%), question generation (5, 5%) or developing recommendations (4, 4%); guideline evaluation and use was the only stage in which people with lived experience were never involved. The method of engagement was as guideline panel members for 95 guidelines (88%), on lived experience panels or advisory groups for 10 (9%), in online surveys for 5 (5%) and in focus groups for 3 guidelines (3%); 17 guidelines (16%) reported multiple methods. The mode of engagement was reported as online in 27 cases (25%), face‐to‐face in 27 (25%) and mixed in 14 (13%); the mode was not reported for 46 guidelines (43%). For 97 guidelines (90%), people with lived experience were asked to decide, advise or vote on recommendations or guideline content, for 64 guidelines (60%) they had governance or approval roles, and for 20 guidelines (19%) they were invited to contribute their views, opinions and experiences. Seven guidelines reported that people with lived experience wrote guideline content (e.g., lay versions); six guidelines reported that they had chaired committees or groups (Table 4).

**TABLE 4:** Nature of lived experience engagement in guideline development for 108 Australian clinical practice guidelines published during 1 January 2014–20 March 2025: Forms of engagement.

Characteristic	Number
Guidelines that reported lived experience engagement	108
Co‐design lived experience engagement	
Yes	11 (10%)
No	97 (90%)
Guideline stages	
Priority setting and topic selection	7 (6%)
Question generation	5 (5%)
Evidence synthesis	3 (3%)
Developing recommendations	4 (4%)
Public consultation	1 (1%)
Dissemination and implementation	2 (2%)
Evaluation and use	0 (0%)
Throughout	98 (91%)
Not reported	3 (3%)
Methods of engagement	
Guideline panel member	95 (88%)
Interviews	2 (2%)
Focus groups	3 (3%)
Workshops/seminars/group consensus	1 (1%)
Delphi/individual consensus study	1 (1%)
Lived experience panel/advisory group	10 (9%)
Online survey	5 (5%)
Other	2 (2%)
Not reported	5 (5%)
Multiple methods of engagement	
Yes	17 (16%)
No	91 (84%)
Mode of engagement	
Online	27 (25%)
Face‐to‐face	27 (25%)
Mixed	14 (13%)
Can't tell	46 (43%)
Assigned tasks/roles	
Chair of committee or group	6 (6%)
Governance/approval role	64 (60%)
Decide, advise or vote on recommendations/guideline content	97 (90%)
Write guideline content (e.g., lay version)	7 (7%)
Contribute views, opinions and experiences	20 (19%)
Feedback	0
Other	2 (2%)
Not reported	3 (3%)
Support provided [31]	
Practical support	49 (45%)
Informal support	17 (16%)
Emotional support	1 (1%)
Remuneration	60 (56%)
Co‐learning and training	17 (16%)
Provide re‐assessment and feedback	0
Manage group dynamics	41 (38%)
Not reported	32 (30%)
Support provided to guideline developers	
Training	2 (2%)
Funding	2 (2%)
Designated staff	6 (6%)
Other	1 (1%)
Not reported	99 (92%)
Level of engagement^a^ [26]	
Inform	0
Consult	1 (1%)
Involve	9 (8%)
Collaborate	99 (92%)
Empower	1 (1%)
Unable to determine	3 (3%)
Evaluation of lived experience engagement	
Yes	5 (5%)

^a^ Inform: to provide the public with balanced and objective information to assist them in understanding the problems, alternatives, opportunities and/or solutions; Consult: to obtain public feedback on analysis, alternatives and/or decisions; Involve: to work directly with the public throughout the process to ensure that public concerns and aspirations are consistently understood and considered; Collaborate: to partner with the public in each aspect of the decision including the development of alternatives and the identification of the preferred solution; Empower: to place the final decision in the hands of the public. Defined by the International Association for Public Participation Australasia [26].

The most frequently reported support for people with lived experience was remuneration (60 of 108 guidelines, 56%), practical support (e.g., plain language meeting papers; 49 guidelines, 45%), management of group dynamics (e.g., chairperson ensured they could actively contribute; 41 guidelines, 38%), informal support (e.g., help with technical queries; 17 guidelines, 16%) and co‐learning and training (e.g., initial orientation and training session; 17 guidelines, 16%). Support provided for guideline developers was not reported in 99 of 108 guidelines (92%); six guidelines reported designated staff for supporting lived experience engagement, two reported training for guideline developers and two reported support funding. The level of engagement according to International Association for Public Participation Australasia definitions [26] was ‘collaborate’ (‘to partner with the public in each aspect of the decision’; 99 of 108 guidelines, 92%), ‘involve’ (‘to work directly with the public throughout the process to ensure that public concerns and aspirations are consistently understood and considered’; nine guidelines), ‘consult’ (‘to obtain public feedback on analysis, alternatives and/or decisions’; one guideline) or ‘empower’ (‘to place the final decision in the hands of the public’; one guideline). Five guidelines reported evaluating lived experience engagement (Table 4).

### 3.3 Examples of More Extensive Lived Experience Engagement

Extensive lived experience engagement was reported for the development of eight guidelines. For the autism assessment and diagnosis guideline [32], people with lived experience were guideline development group members (including one co‐chair), and the developers conducted a large community consultation using online surveys and focus groups. The national stroke living guidelines [33] are developed with a large lived experience panel that reviews and comments on guideline content, two to four members of which join the content development working group when new topics are updated. The guideline for culturally safe and clinical kidney care for First Nations Australians [34] was developed after ‘yarning kidneys’ community consultations across Australia, and three Indigenous people were included as working group members (Table 5).

**TABLE 5:** Eight Australian clinical practice guidelines published during 1 January 2014–20 March 2025 that reported extensive lived experience engagement in guideline development.

Guideline title	Lived experience engagement methods
National guideline for the assessment and diagnosis of autism in Australia [32]	Guideline development group co‐chair had autism; three further lived experience guideline development group members (two people with autism, one parent). Two organisational representatives sat on the reference group, the role of which was to support community consultation. Community consultation consisted of an online survey (805 responses) and 9 focus groups (68 attenders) to learn about the community experiences, views and preferences regarding the assessment and diagnosis of autism.
Australian guideline and calculator for assessing and managing cardiovascular disease risk [35]	Lived experience advisory group (10 people), comprising people with lived experience and family members, including Indigenous representatives, provided feedback on recommendations and guideline content. The chair of the consumer advisory group also sat on the expert steering group and the expert subgroup.
Australian and New Zealand Living Clinical Guidelines for Stroke Management [33]	A 26‐member lived experience panel, comprising stroke survivors and family members, contributed chiefly by email (commenting on recommendations and writing plain language versions of recommendations). For each topic being updated, two to four members join the content development working group to update recommendations. The whole panel was then invited to review and comment on the draft changes.
Clinical practice guidelines for hepatocellular carcinoma surveillance for people at high risk in Australia [36]	Expert advisory group (equivalent to guideline development group) included three lived experience advocates, who, together with one further person with lived experience, were also members of a community reference group, which reviewed the guidelines from a patient perspective.
Recommendations for culturally safe and clinical kidney care in First Nations Australians [34]	Conducted community consultations (yarning kidneys) in 16 communities across Australia (number of participants not reported) to assess community needs and preferences relevant to the proposed guidelines. Three Indigenous people with chronic kidney disease were members of the working group.
Australian Pregnancy Care Guidelines [37] Australian Postnatal Care Guidelines [38]	Online surveys of people with lived experience to identify priority topics for the guideline, a 16‐member lived experience panel to review draft recommendations, 4 co‐chairs and deputy co‐chairs were members of the guidelines leadership group, 2 co‐chairs/deputy co‐chairs each sat on the pregnancy and postnatal panels.
Australian Physical Activity Clinical Practice Guideline for people with moderate to severe traumatic brain injury [39]	Focus groups and interviews with people with lived experience and family members (number of participants not reported) to assess views about the guideline topic. One person with lived experience included in the research programme team and guidelines leadership group; eight people with lived experience and family members included in the guideline development group.

## 4 Discussion

Our scoping review of the extent and nature of lived experience engagement in Australian clinical guideline development included 150 guidelines published during 2014–2025. We found that 108 guidelines (72%) reported involving people with lived experience in their development, of which 61 (56%) reported the participation of one person with lived experience and 14 (13%) of two people with lived experience. Ninety‐eight guidelines (91%) reported lived experience engagement throughout guideline development, primarily as guideline panel members. Very little information about the characteristics of the participating people with lived experience was reported.

The prevalence of lived experience engagement in Australian guideline development during 2014–2025 was much higher than in 2014 (14% [18]); it was also higher than more recently reported for the United States (8% [17]) and Latin America (11% [16]). Lived experience engagement in Australian guideline development may be growing, but it typically consists of one to two people as members of guideline development groups. If only one layperson sits on a group primarily comprised of clinicians, power imbalances can make the experience less than ideal, both for them and for guideline developers [40, 41]. The instances of more extensive engagement we identified are more encouraging. While not appropriate for all guidelines, they provide developers with examples of feasible, effective methods of lived experience engagement.

Nevertheless, it is likely that lived experience engagement in Australian guideline development will continue to be chiefly in the form of development group members. The NHMRC has recently revised its guideline development standards [3]; they now require ‘at least two’ people with lived experience in the guideline development group (previously: one) [3], which should strengthen lived experience engagement. However, based on our professional and personal experience, we believe at least four people should be included, and that consideration be given to their diversity (cultural, gender, age and health literacy) and to the fact that they may require extra support to contribute effectively.

Guideline developers could use our findings to assess their lived experience engagement activities and to find ideas for improving them. The limited reporting of lived experience engagement in Australian guideline development may reflect the limited emphasis on this aspect in widely used reporting tools, such as AGREE II [42]. If amended, these tools could encourage more transparent and comprehensive reporting. A qualitative study of the experiences and perspectives of Australian guideline developers and people with lived experience in guideline development would be useful.

### 4.1 Limitations

Firstly, searching for Australian guidelines is difficult because of the diversity of guideline producers and the absence of a single guideline publishing platform. We believe we identified the vast majority of guidelines, but our sample may be incomplete. Secondly, we did not contact guideline developers to obtain missing information; if we had done so, it which could have yielded a more complete picture, potentially altering some findings. For example, some aspects of lived experience engagement, including remuneration and other support, may not always be reported. Conversely, as we selected guidelines that adhered to our minimum threshold for guideline quality, they may have been more likely to have engaged people with lived experience in their development than other Australian guidelines. Thirdly, although we used the widely accepted US Institute of Medicine definition of ‘guideline’ [2], identifying and categorising unique guidelines is difficult, as they differ in their clinical scope and breadth of topics covered, and sub‐topics or chapters are often published as stand‐alone documents. We grouped guidelines according to the International Classification of Diseases chapters, but this categorisation may not accurately reflect the number of unique guidelines in Australia. Fourthly, we did not assess the overall quality of the included guidelines using a tool such as AGREE II [42], as would be required for assessing the relationship between guideline quality and reported lived experience engagement, but this was not our study aim. Finally, we did not distinguish between laypeople and health professionals as people with lived experience; this information was not usually reported. Including health professionals with lived experience of the topic of the guideline, as people with lived experience can be problematic, particularly if they are the only participating person with lived experience, as they cannot avoid wearing two hats [43].

## 5 Conclusion

Most Australian clinical practice guidelines published during 2014–2025 reported lived experience engagement in the guideline development process, in contrast to a 2014 report. However, extensive lived experience engagement was not reported for the vast majority of guidelines. The engagement of people with lived experience in guideline development needs to be improved to ensure that their values, views and preferences are reflected.

## Author Contributions

Anneliese Synnot: conceptualisation, formal analysis, investigation, methodology, supervision, project administration, validation, writing (original draft), writing (review and editing). Naomi MacPherson: investigation, methodology, validation, writing (review and editing). Thomas Benning: Investigation, Methodology, Validation, Writing (review and editing). Bernard Tso: investigation, methodology, validation, writing (review and editing). Chuyue Wang: investigation, methodology, validation, writing (review and editing). Toni Arfaras: methodology, writing (review and editing). Brian Beh: methodology, writing (review and editing). Vanessa Cullen: methodology, writing (review and editing). Jessica D'Lima: methodology, writing (review and editing). Tony Finneran: methodology, writing (review and editing). David Fry: methodology, writing (review and editing). Michelle King: methodology, writing (review and editing). Alexander Meredith: methodology, writing (review and editing). Jo Muller: methodology, writing (review and editing). Adrian O'Malley: methodology, writing (review and editing). Tari Turner: conceptualisation, methodology, writing (review and editing). Samantha Chakraborty: investigation, methodology, validation, formal analysis, supervision, writing (review and editing).

## Funding

The authors have nothing to report.

## Disclosure

Not commissioned; externally peer reviewed.

## Conflicts of Interest

The authors declare no conflicts of interest.

## Supporting information


**Data S1:** mja270132‐sup‐0001‐supinfo.pdf.

## Data Availability

The study data can be accessed by contacting the corresponding author.
