# Supplementary material for: The Extent and Nature of Lived Experience Engagement in the Development of Australian Clinical Practice Guidelines, 2014–2025: A Scoping Review
Source: Med J Aust. 2026 Feb 2;224(2):e70132. doi: 10.5694/mja2.70132 (PMC12863021; doi:10.5694/mja2.70132)
Supplement: Supplementary file 1 — Data S1: mja270132‐sup‐0001‐supinfo.pdf. [file MJA2-224-0-s001.pdf]

Supporting Information

Supplementary methods and results

This appendix was part of the submitted manuscript and has been peer reviewed.
It is posted as supplied by the authors.

Appendix to: A. Synnot, N. MacPherson, T. Benning, et al. The Extent and Nature of Lived Experience Engagement in the Development of Australian Clinical Practice Guidelines, 2014–2025: A Scoping Review. *Med J Aust* 2026; doi: 10.5694/mja2.70132.

Supplementary methods

Table S1. Data extraction template

Year of most recent publication	YYYY
Guideline topic (https://icd.who.int/en)	International Classification of Disease 11 chapters
NHMRC approval	Yes or No
Author (guideline group /organisation / publisher)	Free text
Looked for evidence of patient values preferences and experiences (for any question)?	Yes or No
Conducted public consultation phase?	Yes or No
If yes, did they specifically seek out lived experience input in the public consultation phase?	Yes or No
Involved people with lived experience (at least 1) in any stage / way in guideline development	Yes or No
Involved people with lived experience in more than one way?	Yes or No
Involved people with lived experience in governance / approval?	Yes or No
Lived experience involvement in planning methods for lived experience involvement	Yes or No
Recruitment approach (see Key)	Not reported
	Open - Fixed
	Open - Flexible
	Closed - Invitation
	Closed - Existing group
	Closed - Purposive sampling
Recruitment source	Nor reported
	Lived experience groups
	Patient records from healthcare providers
	Individual patients
	Contacts of researchers or guideline developers
Guideline stages when people with lived experience were engaged	Priority setting and topic selection
	Question generation
	Evidence Synthesis
	Developing recommendations
	Public Consultation
	Dissemination and implementation
	Evaluation and Use
	Throughout
	Not reported
Number of people with lived experience	[Number] or not reported
Type of people with lived experience	Not reported
	Person with lived experience / patient
	Family member
	Advocate
	Organisational representative
PROGRESS-PLUS characteristics	Not reported
	Place of residence (regional, remote, homeless)

	Race/culture/ethnicity/language
	Occupation (unemployed/high risk environment)
	Gender and sex
	Religion
	Education (limited)
	Socioeconomic status (limited income)
	Social capital (social isolation and limited networks)
	Age
	Sexual orientation
	Disability
Other demographic characteristics (e.g. disease severity)	Free text
Diversity<br>-Efforts to boost diversity of lived experience input?<br>-Outcome of these efforts	Free text
Methods of engaging with people with lived experience	Guideline panel member
	Interviews
	Focus groups
	Workshops/seminars/group consensus
	Delphi/individual consensus study
	Other
	Not reported
Mode of engagement	Online
	Face-to-face
	Mixed
	Can't tell
Tasks/roles assigned to people with lived experience	Chair of committee or group
	Oversight role
	Decide, advise or vote on recommendations
	Write guideline content (e.g. lay version)
	Contribute views, opinions and experiences
	Provide feedback
	Other
	Not reported
Support provided to people with lived experience	Not reported
	Practical support
	Informal support
	Emotional support
	Remuneration
	Co-learning and training
	Provide re-assessment and feedback
	Managing group dynamics
Support provided to guideline developers in working with people with lived experience	Not reported
	Training
	Funding
	Designated staff
	Other

Level of engagement (see key)	Inform
	Consult
	Involve
	Collaborate
	Empower
	Unable to determine
Evaluation of lived experience engagement	Yes or No
Evaluation methods of lived experience engagement<br>- Aim of evaluation<br>- Who participated?<br>- What methods?<br>- How were the results used?	Free text
Source used	Free text
Additional references / citations	Free text

Key

Item: Recruitment approach[1]

- Open = ‘Open’ recruitment refers to providing opportunities for involvement through advertisement to the general population, allowing anyone to volunteer to get involved.
    - Fixed = Open recruitment may result in ‘fixed’ membership, where, once group members had volunteered, the membership remains the same, or
    - Flexible = in ‘flexible’ membership, where different people attend different events or contribute to different activities.
- Closed = Conversely, ‘closed’ (or ‘targeted’) recruitment strategies focus on inviting only specific people to participate.
    - Invitation = Closed strategies include invitation of known individuals or recognized experts,
    - Existing group = recruitment from membership of an existing group,
    - Purposive sampling = or purposive sampling to achieve representation of people with key pre-determined characteristics, experience or expertise.

Item: Support provided to people with lived experience[2]

- Practical support: Agreeing reasonable adjustments (e.g., coloured paper for people with dyslexia, or changing the environment to increase accessibility); providing meeting papers in advance; explaining or avoiding medical jargon
- Informal support: Key contact person for technical work or public involvement; peer-support; pre-meetings and debrief meetings to provide support and feedback; check-in calls or emails.
- Emotional support: Check-in calls or emails; help the individual identify coping mechanisms and support networks; foster relationships with other patient/public members (buddying)
- Remuneration: Consider the type of compensation, such as financial remuneration, vouchers, reimburse travel or accommodation expenses, carer or childcare costs, free training and skill development.
- Co-learning and training:
    - Training: Develop or signpost to existing training, workshops, seminars, online modules, or courses on guideline development processes and methods (e.g., GRADE); research methods and terminology; technical skills; group participation and relationship building skills.
    - Co-learning: implement opportunities for co-learning with other panel members throughout the guideline development lifecycle e.g., presentations at the start of meetings on GRADE; free access to online journals. Buddying/peer-support, pre-meets and debriefs with technical staff, and providing feedback are opportunities to encourage co-learning and individual development in their role.
- Provide re-assessment and feedback: Implement opportunities (e.g., pre-meets, debriefs, experience surveys and informal meetings) to provide constructive feedback on the person’s performance in their role. Can facilitate co-learning and development of skills. Ensures patient/public members feel valued and supported which helps to foster continuous engagement.
- Managing group dynamics: Understanding group dynamics and how to manage power dynamics e.g., highlighting the importance of patient and public involvement through a presentation or using storytelling techniques can foster the panel’s understanding, empathy and importance of the patient journey. Chair training is crucial for understanding and management group dynamics.

Item: Level of engagement[3]

- Inform: to provide the public with balanced and objective information to assist them in understanding the problems, alternatives, opportunities and/ or solutions
- Consult: to obtain public feedback on analysis, alternatives and/ or decisions
- Involve: to work directly with the public throughout the process to ensure that public concerns and aspirations are consistently understood and considered
- Collaborate: to partner with the public in each aspect of the decision including the development of alternatives and the identification of the preferred solution
- Empower: to place the final decision in the hands of the public

References

1 Pollock A, Campbell P, Struthers C, et al. Development of the ACTIVE framework to describe stakeholder involvement in systematic reviews. J Health Serv Res Policy 2019; 24: 245-255.

2 Scott S, Cowl J, Graham CSK, et al. Recruitment and support. GIN public toolkit: patient and public involvement in guidelines, undated, accessed Sept 2025, https://g-i-n.net/chapter/recruitment-and-support

3 International Association for Public Participation Australasia. IAP2 public participation spectrum. Updated 13 June 2025, accessed June 2025, https://engagementinstitute.org.au/resources/iap2-public-participation-spectrum

Supplementary results

Figure S1. PRISMA study selection flow chart

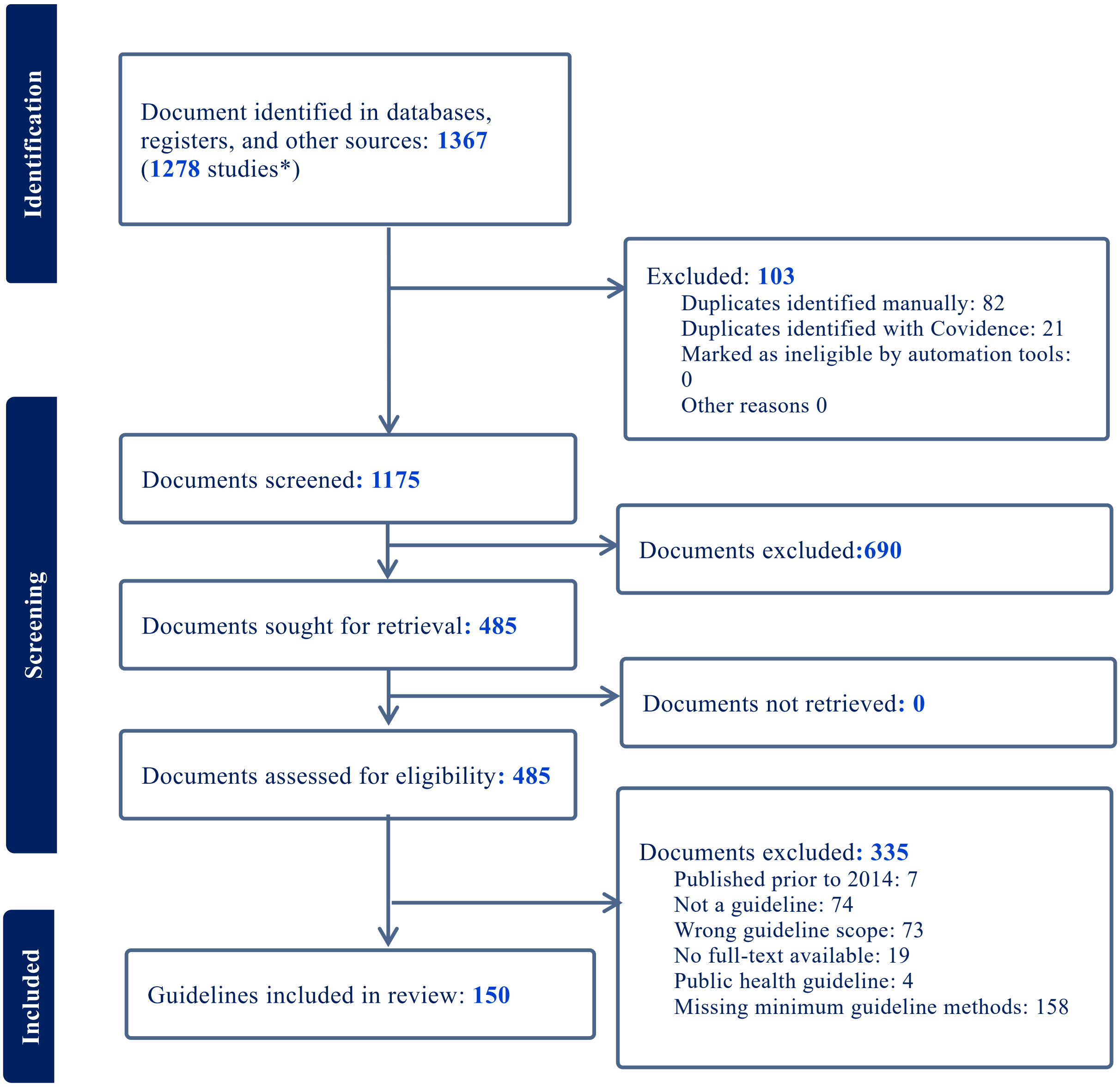

* Some guidelines were reported in multiple publications or published in chapters, which were subsequently merged into single studies.

Table S2. The 150 included guidelines

1. Richards BL, Whittle S, Buchbinder R, Barrett C, Lynch N, Major G, Littlejohn G, Taylor A and Zochling J. Australian and New Zealand evidence-based recommendations for pain management by pharmacotherapy in adult patients with inflammatory arthritis Int J Rheum Dis 2014 17: 738-748

2. Hay P, Chinn D, Forbes D, Madden S, Newton R, Sugenor L, Touyz S, Ward W; Royal Australian and New Zealand College of Psychiatrists.. Royal Australian and New Zealand College of Psychiatrists clinical practice guidelines for the treatment of eating disorders Aust NZ J Psychiatry 2014 48:977-1008

3. Langham RG, Bellomo R, D'Intini V, Endre Z, Hickey BB, McGuinness S, Phoon RK, Salamon K, Woods J, Gallagher MP; Kidney Health Australia Caring for Australasians with Renal Impairment; Kidney Disease Improving Global Outcomes. KHA-CARI guideline: KHA-CARI adaptation of the KDIGO Clinical Practice Guideline for Acute Kidney Injury. Nephrology 2014 19:261-265

4. Stillbirth Foundation Australia and Johanna Briggs Institute. Caring for families experiencing stillbirth, 2014. https://ranzcog.edu.au/wp-content/uploads/Caring-for-Families-Experiencing-Stillbirth-Part-1.pdf (viewed Sept 2025)

5. McCombie SP, Thyer I, Corcoran NM, Rowling C, Dyer J, Le Roux A, Kuan M, Wallace DM, Hayne D. The conservative management of renal trauma: a literature review and practical clinical guideline from Australia and New Zealand. BJU Int. 2014 114 Suppl 1:13-21

6. Caring for Australians and New Zealanders with Kidney Impairment (CARI). Peritonitis Treatment and Prophylaxis, 2014. https://www.cariguidelines.org/guidelines/dialysis/peritonitis-treatment-and-prophylaxis/ (viewed Sept 2025)

7. Cancer Council Australia. Australian clinical practice guidelines for the diagnosis and management of Barrett's esophagus and early esophageal adenocarcinoma, 2014. https://www.cancer.org.au/assets/pdf/9-august-2020 (viewed Sept 2025)

8. The Royal Australian College of General Practitioners. Smoking, nutrition, alcohol, physical activity (SNAP): A population health guide to behavioural risk factors in general practice, 2015. https://www.racgp.org.au/clinical-resources/clinical-guidelines/key-racgp-guidelines/view-all-racgp-guidelines/snap (viewed Sept 2025)

9. McTaggart S, Danchin M, Ditchfield M, Hewitt I, Kausman J, Kennedy S, Trnka P, Williams G; Kidney Health Australia - Caring for Australasians with Renal Impairment. KHA-CARI guideline: Diagnosis and treatment of urinary tract infection in children Nephrology 2015 20:55-60

10. National Blood Authority. Patient Blood Management Guidelines: Module 5 Obstetrics and Maternity, 2015. https://www.blood.gov.au/module-5-obstetrics-and-maternity-patient-blood-management-guidelines (viewed Sept 2025)

11. The Thoracic Society of Australia and New Zealand. Oxygen guidelines for acute oxygen use in adults: Clinical practice guideline "Swimming between the flags", 2015. https://thoracic.org.au/wp-content/uploads/2021/06/TSANZ-AcuteOxygen-Guidelines-2016-web.pdf (viewed Sept 2025)

12. Chaves NJ, Paxton G, Biggs BA, Thambiran A, Smith M, Williams J, Gardiner J, Davis JS; on behalf of the Australasian Society for Infectious Diseases and Refugee Health Network of Australia Guidelines writing group. Recommendations for comprehensive post-arrival health assessment for people from refugee-like backgrounds, 2016. https://www.rch.org.au/uploadedFiles/Main/Content/immigranthealth/ASID-RHeaNA%20screening%20guidelines.pdf (viewed Sept 2025)

13. Carter G, Page A, Large M, Hetrick S, Milner AJ, Bendit N, Walton C, Draper B, Hazell P, Fortune S, Burns J, Patton G, Lawrence M, Dadd L, Dudley M, Robinson J, Christensen H.. Royal Australian and New Zealand College of Psychiatrists clinical practice guideline for the management of deliberate self-harm. Aust NZ J Psychiatry 2016 50:939-1000

14. Galletly C, Castle D, Dark F, Humberstone V, Jablensky A, Killackey E, Kulkarni J, McGorry P, Nielssen O, Tran N. Royal Australian and New Zealand College of Psychiatrists clinical practice guidelines for the management of schizophrenia and related disorders. Aust NZ J Psychiatry 2016 50:410-72.

15. Chew DP, Scott IA, Cullen L, French JK, Briffa TG, Tideman PA, Woodruffe S, Kerr A, Branagan M, Aylward PE; NHFA/CSANZ ACS Guideline 2016 Executive Working Group:. National Heart Foundation of Australia & Cardiac Society of Australia and New Zealand: Australian clinical guidelines for the management of acute coronary syndromes. Heart Lung Circ 2016 25:895-951

16. Gabb GM, Mangoni AA, Anderson CS, Cowley D, Dowden JS, Golledge J, Hankey GJ, Howes FS, Leckie L, Perkovic V, Schlaich M, Zwar NA, Medley TL, Arnolda L. Guideline for the diagnosis and management of hypertension in adults Med J Aust 2016 205: 85-9

17. Guideline Adaptation Committee. Clinical practice guidelines and principles of care for people with dementia, 2016. https://cdpc.sydney.edu.au/wp-content/uploads/2019/06/CDPC-Dementia-Guidelines_WEB.pdf (viewed Sept 2025)

18. National Blood Authority. Module 6 Neonatal and paediatrics: patient blood management guidelines, 2016. available at https://www.blood.gov.au/module-6-neonatal-and-paediatrics-patient-blood-management-guidelines (viewed Sept 2025)

19. Royal Australian and New Zealand College of Obstetricians and Gynaecologists. Diagnosis of gestational diabetes mellitus (GDM), 2017. https://ranzcog.edu.au/wp-content/uploads/Diagnosis-Gestational-Diabetes-Mellitus.pdf (viewed Sept 2025)

20. Cancer Council Australia, Clinical Oncology Society of Australia. COSA guidelines for the safe prescribing, dispensing and administration of systemic cancer therapy, 2017. https://app.magicapp.org/#/guideline/8085 (viewed Sept 2025)

21. Alison JA, McKeough ZJ, Johnston K, McNamara RJ, Spencer LM, Jenkins SC, Hill CJ, McDonald VM, Frith P, Cafarella P, Brooke M, Cameron-
Tucker HL, Candy S, Cecins N, Chan ASL, Dale M, Dowman LM, Granger C, Halloran S, Jung P, LeeA, Leung R, Matulick T, Osadnik C, Roberts M, Walsh J, Wootton S, Holland AE and on behalf of the Lung Foundation Australia and the Thoracic Society of Australia and New Zealand Australian and New Zealand Pulmonary Rehabilitation Guidelines Respirology 2017 22: 800–819

22. Andrews G, Bell C, Boyce P, Gale C, Lampe L, Marwat O, Rapee R, Wilkins G. Royal Australian and New Zealand College of Psychiatrists clinical practice guidelines for the treatment of panic disorder, social anxiety disorder and generalised anxiety disorder Australian & New Zealand Journal of Psychiatry 2018 52:1109-1172

23. Royal Australian College of General Practitioners. Guideline for the management of knee and hip osteoarthritis 2nd edition, 2018. https://www.racgp.org.au/getattachment/71ab5b77-afdf-4b01-90c3-04f61a910be6/Guideline-for-the-management-of-knee-and-hip-osteoarthritis.aspx (viewed Sept 2025)

24. Prostate Cancer Foundation Australia and Cancer Council Australia. PSA testing and early management of test-detected prostate cancer: Clinical practice guidelines, 2018. https://www.pcfa.org.au/awareness/for-healthcare-professionals/clinical-practice-guidelines-on-psa-testing/ (viewed Sept 2025)

25. Reeve E, Farrell B, Thompson W, Herrmann N, Sketris I, Magin P, Chenoweth L, Gorman M, Quirke L, Bethune G, Forbes F, Hilmer S. Evidence-based Clinical Practice Guideline for Deprescribing Cholinesterase Inhibitors and Memantine, 2018 http://sydney.edu.au/medicine/cdpc/resources/deprescribing-guidelines.php (viewed Sept 2025)

26. Royal Australian and New Zealand College of Obstetricians and Gynaecologists. Management of gestational trophoblastic disease. 2017. https://ranzcog.edu.au/wp-content/uploads/Management-Gestational-Trophoblastic-Disease.pdf (viewed Sept 2025)

27. Cancer Council. Clinical practice guidelines for the treatment of lung cancer, 2017. https://www.cancer.org.au/clinical-guidelines/lung-cancer/lung-cancer-treatment (viewed Sept 2025)

28. Atherton JJ, Sindone A, De Pasquale CG, Driscoll A, MacDonald PS, Hopper I, Kistler P, Briffa TG, Wong J, Abhayaratna WP, Thomas L, Audehm R, Newton PJ, O'Loughlin J, Connell C, Branagan M. 2018. National Heart Foundation of Australia and Cardiac Society of Australia and New Zealand: Australian clinical guidelines for the management of heart failure. Med J Aust 2018 209: 363-369

29. Royal Australian and New Zealand College of Obstetricians and Gynaecologists. Progesterone support of the luteal phase and in the first trimester, 2018. https://ranzcog.edu.au/wp-content/uploads/2022/05/Progesterone-Support-of-the-Luteal-Phase-and-in-the-First-Trimester.pdf (viewed Sept 2025)

30. Cancer Council Australia Surveillance Colonoscopy Guidelines Working Party. Clinical Practice Guidelines for Surveillance Colonoscopy, 2018. https://cancerwa.asn.au/assets/public/2022/07/2020-11-26-Short-form-guidelines-Colonoscopy-surveillance-Summary-of-recommendations-Mar19.pdf (viewed Sept 2025)

31. National Heart Foundation of Australia and the Cardiac Society of Australia and New Zealand Atrial Fibrillation Guideline Working Group; Brieger D, Amerena J, Attia J, Bajorek B, Chan KH, Connell C, Freedman B, Ferguson C, Hall T, Haqqani H, Hendriks J, Hespe C, Hung J, Kalman JM, Sanders P, Worthington J, Yan TD, Zwar N. 2018. National Heart Foundation of Australia and the Cardiac Society of Australia and New Zealand: Australian Clinical Guidelines for the Diagnosis and Management of Atrial Fibrillation. Heart Lung Circ 2018 27:1209-1266

32. Medley TL, Miteff C, Andrews I, Ware T, Cheung M, Monagle P, Mandelstam S, Wray A, Pridmore C, Troedson C, Dale RC, Fahey M, Sinclair A, Walsh P, Stojanovski B, Mackay MT. Australian Clinical Consensus Guideline: the diagnosis and acute management of childhood stroke. Int J Stroke 2019 14:94-106

33. Royal Australian and New Zealand College of Obstetricians and Gynaecologists Vaginal 'rejuvenation' and cosmetic vaginal procedures, 2019. https://ranzcog.edu.au/wp-content/uploads/Vaginal-Rejuvenation-Cosmetic-Vaginal-Procedures.pdf (viewed Sept 2025)

34. Jardine, M., Commons, R.J., de Zoysa, J.R., Wong, M.G., Gilroy, N., Green, J., Henderson, B., Stuart, R.L., Tunnicliffe, D.J., van Eps, C. and Athan, E. Kidney Health Australia - Caring for Australasians with Renal Impairment guideline recommendations for infection control for haemodialysis units 2019 Nephrology, 24: 951-957

35. Mazza D, Brijnath B, Chakraborty, SP and the Guideline Development Group. Clinical guideline for the diagnosis and management of work-related mental health conditions in general practice. 2019. Melbourne: Monash University, https://www.monash.edu/medicine/sphpm/general-practice/engagement/clinical-guidelines (viewed Sept 2025)

36. Caring for Australians and New Zealanders with Kidney Impairment (CARI). Kidney biopsy, 2019. https://www.cariguidelines.org/guidelines/chronic-kidney-disease/kidney-biopsy/ (viewed Sept 2025)

37. O'Brien S, Borland ML, Cotterell E, Armstrong D, Babl F, Bauert P, Brabyn C, Garside L, Haskell L, Levitt D, McKay N, Neutze J, Schibler A, Sinn K, Spencer J, Stevens H, Thomas D, Zhang M, Oakley E, Dalziel SR; Paediatric Research in Emergency Departments International Collaborative (PREDICT) Network, Australasia. Australasian bronchiolitis guideline J Paediatr Child Health 2019 55:42-53

38. Royal Australian and New Zealand College of Obstetricians and Gynaecologists. Vasa Praevia (C-Obs 47), 2019. https://ranzcog.edu.au/wp-content/uploads/Vasa-Praevia.pdf (viewed Sept 2025)

39. Royal Australian and New Zealand College of Obstetricians and Gynaecologists. C-Gyn 18 Guidelines for Human Papillomavirus (HPV) Vaccine, 2019. https://ranzcog.edu.au/wp-content/uploads/Guidelines-HPV-Vaccine.pdf (viewed Sept 2025)

40. Royal Australian and New Zealand College of Obstetricians and Gynaecologists. Intrapartum Fetal Surveillance (4th edition), 2019. https://ranzcog.edu.au/wp-content/uploads/Intrapartum-Fetal-Surveillance.pdf (viewed Sept 2025)

41. Royal Australian and New Zealand College of Obstetricians and Gynaecologists. Tamoxifen and the endometrium, 2019. https://ranzcog.edu.au/wp-content/uploads/Tamoxifen-and-the-Endometrium.pdf (viewed Sept 2025)

42. Royal Australian and New Zealand College of Obstetricians and Gynaecologists. Instrumental vaginal birth, 2020. https://ranzcog.edu.au/wp-content/uploads/Instrumental-Vaginal-Birth.pdf (viewed Sept 2025)

43. Cancer Council Australia. Clinical practice guidelines for the diagnosis and management of melanoma, 2020. https://www.cancer.org.au/assets/pdf/archived-diagnosis-and-management-of-melanoma-guidelines-22-april-2021 (viewed Sept 2025)

44. University of Sydney. Clinical practice guideline for the management of communication and swallowing in children diagnosed with childhood brain tumour or leukaemia, 2020. https://www.cancer.org.au/clinical-guidelines/communication-swallowing-childhood-brain-tumour-leukaemia (viewed Sept 2025)

45. Royal Australian and New Zealand College of Obstetricians and Gynaecologists. Managing menopausal symptoms, 2020. https://ranzcog.edu.au/wp-content/uploads/Managing-Menopausal-Symptoms.pdf (viewed Sept 2025)

46. Royal Australian and New Zealand College of Obstetricians and Gynaecologists. Management of Hepatitis C in pregnancy, 2020. https://ranzcog.edu.au/wp-content/uploads/Management-Hepatitis-C-Pregnancy.pdf (viewed Sept 2025)

47. Royal Australian and New Zealand College of Obstetricians and Gynaecologists. Management of Hepatitis B in pregnancy, 2019. https://ranzcog.edu.au/wp-content/uploads/2022/05/Management-of-Hepatitis-B-in-pregnancy-C-Obs-50.pdf (viewed Sept 2025)

48. Royal Australian and New Zealand College of Obstetricians and Gynaecologists. Uterine artery embolization for the treatment of uterine fibroids, 2020. https://ranzcog.edu.au/wp-content/uploads/Uterine-Artery-Embolisation-Fibroids.pdf (viewed Sept 2025)

49. Royal Australian and New Zealand College of Obstetricians and Gynaecologists. Exercise during pregnancy, 2020. https://ranzcog.edu.au/wp-content/uploads/Exercise-During-Pregnancy-Guidance.pdf (viewed Sept 2025)

50. Royal Australian and New Zealand College of Obstetricians and Gynaecologists. Vaginal screening after hysterectomy in Australia, 2020. https://ranzcog.edu.au/wp-content/uploads/2022/05/Vaginal-screening-after-hysterectomy-in-Australia-C-Gyn-8.pdf (viewed Sept 2025)

51. Royal Australian and New Zealand College of Obstetricians and Gynaecologists. Polypropylene vaginal mesh implants for vaginal prolapse, 2021. https://ranzcog.edu.au/wp-content/uploads/Polypropylene-Vaginal-Mesh-Implants-Vaginal-Prolapse.pdf (viewed Sept 2025)

52. Royal Australian and New Zealand College of Obstetricians and Gynaecologists. Term Prelabour Rupture of Membranes (Term PROM), 2021 https://ranzcog.edu.au/wp-content/uploads/Term-PROM.pdf (viewed Sept 2025)

53. Royal Australian and New Zealand College of Obstetricians and Gynaecologists. Management of monochorionic twin pregnancy, 2021. https://ranzcog.edu.au/wp-content/uploads/Management-Monochorionic-Twin-Pregnancy.pdf (viewed Sept 2025)

54. Royal Australian and New Zealand College of Obstetricians and Gynaecologists. Use of the Veress needle to obtain pneumoperitoneum prior to laparoscopy, 2021. https://ranzcog.edu.au/wp-content/uploads/Use-of-Veress-Needle-Pneumoperitoneum-Laparoscopy.pdf (viewed Sept 2025)

55. Royal Australian and New Zealand College of Obstetricians and Gynaecologists. Mental Health Care in the Perinatal Period, 2021. https://ranzcog.edu.au/wp-content/uploads/Mental-Health-Care-Perinatal-Period.pdf (viewed Sept 2025)

56. Malhi GS, Bell E, Bassett D, Boyce P, Bryant R, Hazell P, Hopwood M, Lyndon B, Mulder R, Porter R, Singh AB, Murray G.. The 2020 Royal Australian and New Zealand College of Psychiatrists clinical practice guidelines for mood disorders. Aust NZ J Psychiatry 2021 55:7-117

57. Babl FE, Tavender E, Ballard DW, Borland ML, Oakley E, Cotterell E, Halkidis L, Goergen S, Davis GA, Perry D, Anderson V, Barlow KM, Barnett P, Bennetts S, Bhamjee R, Cole J, Craven J, Haskell L, Lawton B, Lithgow A, Mullen G, O'Brien S, Paproth M, Wilson CL, Ring J, Wilson A, Leo GS, Dalziel SR; Paediatric Research in Emergency Departments International Collaborative (PREDICT). Australian and New Zealand Guideline for Mild to Moderate Head Injuries in Children Emerg Med Australas 2021 33:214-231

58. Cancer Council. Clinical practice guidelines for keratinocyte cancer, 2021. https://app.magicapp.org/#/guideline/n3QxOj (viewed Sept 2025)

59. National Clinical Evidence Taskforce. Australian guidelines for the clinical care of people with COVID-19, 2022. https://app.magicapp.org/#/guideline/L4Q5An/rec/jboeKl (viewed Sept 2025)

60. Royal Australian and New Zealand College of Obstetricians and Gynaecologists Cross-border reproductive care, 2021. https://ranzcog.edu.au/wp-content/uploads/Cross-Border-Reproductive-Care.pdf (viewed Sept 2025)

61. Royal Australian and New Zealand College of Obstetricians and Gynaecologists. Water immersion during labour and birth, 2021. https://ranzcog.edu.au/wp-content/uploads/Water-Immersion-Labour-Birth.pdf (viewed Sept 2025)

62. Ralph AP, Noonan S, Wade V, Currie BJ. The 2020 Australian guideline for prevention, diagnosis and management of acute rheumatic fever and rheumatic heart disease. Med J Aust 2021 214:220-227

63. Royal Australian and New Zealand College of Obstetricians and Gynaecologists. Measurement of cervical length for prediction of preterm birth, 2021. https://ranzcog.edu.au/wp-content/uploads/Measurement-Cervical-Length-Preterm-Birth.pdf (viewed Sept 2025)

64. Royal Australian and New Zealand College of Obstetricians and Gynaecologists. Diagnosis and management of suspected fetal macrosomia, 2021. https://ranzcog.edu.au/wp-content/uploads/Diagnosis-Management-Suspected-Fetal-Macrosomia.pdf (viewed Sept 2025)

65. Royal Australian and New Zealand College of Obstetricians and Gynaecologists. Reproductive health for women with an intellectual disability, 2021. https://ranzcog.edu.au/wp-content/uploads/Reproductive-Health-Intellectual-Disability.pdf (viewed Sept 2025)

66. Royal Australian and New Zealand College of Obstetricians and Gynaecologists Management of breech presentation, 2021. https://ranzcog.edu.au/wp-content/uploads/Management-Breech-Presentation.pdf (viewed Sept 2025)

67. Royal Australian and New Zealand College of Obstetricians and Gynaecologists. Investigation of intermenstrual and postcoital bleeding, 2021. https://ranzcog.edu.au/wp-content/uploads/Investigation-Intermenstrual-Postcoital-Bleeding.pdf (viewed Sept 2025)

68. Leach AJ, Morris PS, Coates HL, Nelson S, O'Leary SJ, Richmond PC, Gunasekera H, Harkus S, Kong K, Brennan-Jones CG, Brophy-Williams S, Currie K, Das SK, Isaacs D, Jarosz K, Lehmann D, Pak J, Patel H, Perry C, Reath JS, Sommer J, Torzillo PJ. Otitis media guidelines for Australian Aboriginal and Torres Strait Islander children: summary of recommendations. Med J Aust. 2021214:228-233

69. Haber PS, Riordan BC. Guidelines for the Treatment of Alcohol Problems (4th edition), 2021. https://alcoholtreatmentguidelines.com.au/pdf/guidelines-for-the-treatment-of-alcohol-problems.pdf (viewed Sept 2025)

70. Royal Australian and New Zealand College of Obstetricians and Gynaecologists Management of Postpartum Haemorrhage (PPH), 2021. https://ranzcog.edu.au/wp-content/uploads/Management-Postpartum-Haemorrhage.pdf (viewed Sept 2025)

71. Royal Australian and New Zealand College of Obstetricians and Gynaecologists Prevention, detection, and management of subgaleal haemorrhage in the newborn, 2021. https://ranzcog.edu.au/wp-content/uploads/Subgaleal-Haemorrhage.pdf (viewed Sept 2025)

72. Phoenix Australia. Australian Guidelines for the Prevention and Treatment of Acute Stress Disorder, 2021. Posttraumatic Stress Disorder and Complex PTSD, available at https://www.phoenixaustralia.org/australian-guidelines-for-ptsd/ (viewed Sept 2025)

73. Greenham M, Knight S, Rodda J, Scheinberg A, Anderson V, Fahey MC, Mackay MT; Victorian Subacute Childhood Stroke Advisory Committee. Australian clinical consensus guideline for the subacute rehabilitation of childhood stroke Int J Stroke 2021 16:311-320

74. Lazzarini PA, Raspovic A, Prentice J, Commons RJ, Fitridge RA, Charles J, Cheney J, Purcell N, Twigg SM, on behalf of the Australian Diabetes-related Foot Disease Guidelines & Pathways Project. Australian evidence-based guidelines for diabetes-related foot disease; version 1.0, 2021. https://www.diabetesfeetaustralia.org/wp-content/uploads/2021/12/2021-Australian-guidelines-for-diabetes-related-foot-disease-V1.0191021.pdf (viewed Sept 2025)

75. Clements, W., Brown, N., Buckley, B., Rogan, C., Kok, H.K. and Liang, E. Quality care guidelines for uterine artery embolisation in women with symptomatic uterine fibroids in Australia and New Zealand: According to the AGREE-II checklist and endorsed by the Interventional Radiology Society of Australasia. J Med Imaging Radiat Oncol 2022 66: 819-882

76. Brown WJ, Hayman M, Haakstad LAH, Lamerton T, Mena GP, Green A, Keating SE, Gomes GAO, Coombes JS, Mielke GI Australian guidelines for physical activity in pregnancy and postpartum. J Sci Med Sport 2022 25:511-519

77. Caring for Australians and New Zealanders with Kidney Impairment (CARI). Recommendations for culturally safe and clinical kidney care in First Nations Australians, 2022. https://www.cariguidelines.org/first-nations-australian-guidelines/ (viewed Sept 2025)

78. Royal Australian College of General Practitioners. Abuse and violence - Working with our patients in general practice, 2022. https://www.racgp.org.au/getattachment/4ab6102c-67d9-4440-9398-a3ae759164ef/Abuse-and-violence-Working-with-our-patients-in-general-practice.aspx (viewed Sept 2025)

79. Australian and New Zealand College of Anaesthetists and the Faculty of Pain Medicine. Updated guideline on equipment to manage difficult airways: Australian and New Zealand College of Anaesthetists, 2022. https://www.anzca.edu.au/getattachment/02fe1a4c-14f0-4ad1-8337-c281d26bfa17/PS56-Guideline-on-equipment-to-manage-difficult-airways (viewed Sept 2025)

80. Clinical Oncology Society of Australia. COSA guidelines for teleoncology, 2022. https://app.magicapp.org/#/guideline/nYYb4n (viewed Sept 2025)

81. Clinical Oncology Society of Australia. COSA guidelines for fertility preservation for people with cancer, 2022. https://www.cancer.org.au/clinical-guidelines/cancer-fertility-preservation (viewed Sept 2025)

82. Royal Australian and New Zealand College of Obstetricians and Gynaecologists. Power morcellation at Minimally Invasive Procedures, 2022. https://ranzcog.edu.au/wp-content/uploads/Power-Morcellation-Minimally-Invasive-Procedures.pdf (viewed Sept 2025)

83. Langford AV, Schneider CR, Lin CWC, Bero L, Blyth FM, Doctor JN, Holliday S, Jeon YH, Moullin JC, Murnion B, Nielsen S, Osman R, Penm J, Reeve E, Reid S, Wale J, Gnjidic D. Evidence-based Clinical Practice Guideline for Deprescribing Opioid Analgesics, 2022. https://www.opioiddeprescribingguideline.com/guideline (viewed Sept 2025)

84. Royal Australian and New Zealand College of Obstetricians and Gynaecologists. Timing of planned caesarean section at term, 2022. https://ranzcog.edu.au/wp-content/uploads/Timing-Planned-Caesarean-Section.pdf (viewed Sept 2025)

85. Royal Australian and New Zealand College of Obstetricians and Gynaecologists. Management of Obesity in Pregnancy, 2022. https://ranzcog.edu.au/wp-content/uploads/Management-Obesity-Pregnancy.pdf (viewed Sept 2025)

86. Royal Australian and New Zealand College of Obstetricians and Gynaecologists. Subclinical hypothyroidism and hypothyroidism in pregnancy, 2022. https://ranzcog.edu.au/wp-content/uploads/Hypothyroidism.pdf (viewed Sept 2025)

87. Simon Bell, Ravi Bhat, Sue Brennan, Malcolm Clark, Megan Corlis, Christopher Etherton-Beer, Susan Field, Julia Gilmartin-Thomas, Terrence Haines, Sarah Hilmer, Leanne Jack, Alison Kitson, Constance Kourbelis, Sue Kurrle, Dina Logiudice, Steve Macfarlane, Tuan Nguyen, Amy Page, Dimity Pond, Davina Porock, Tara Quirke, Velandai Srikanth, Andrew Stafford, Jane Thompson, Edwin Tan, Jacqueline Wesson (Guideline Development Group). Clinical Practice Guidelines for the Appropriate Use of Psychotropic Medications in People Living with Dementia and in Residential Aged Care: Summary of Recommendations and Good Practice Statements, 2022. https://www.monash.edu/__data/assets/pdf_file/0005/3657128/240424_Clinical-Pracitcie-Guidelines-for-the-Appropriate-Use-of-Psychotorpic-Medications-in-People-Living-with-Dementia-and-in-Residential-Aged-Care.pdf (viewed Sept 2025)

88. Royal Australian and New Zealand College of Obstetricians and Gynaecologists. Routine antenatal assessment in the absence of pregnancy complications, 2022. https://ranzcog.edu.au/wp-content/uploads/Routine-Antenatal-Assessment.pdf (viewed Sept 2025)

89. Australasian ADHD Professionals Association. Australian Evidence-based clinical guideline for attention deficit hyperactivity disorder (ADHD), 2022. https://adhdguideline.aadpa.com.au/ (viewed Sept 2025)

90. Perinatal Society of Australia and New Zealand and Centre of Research Excellence Stillbirth. Clinical practice guideline for the care of women with decreased fetal movements with a singleton pregnancy from 28 weeks' gestation, 2022. https://learn.stillbirthcre.org.au/wp-content/uploads/2023/05/DFM_Clinical-Practice-Guideline_V2.5_Mar2023.pdf (viewed Sept 2025)

91. Royal Australian and New Zealand College of Obstetricians and Gynaecologists. C-Gyn 25 Managing the adnexa at the time of hysterectomy for benign gynaecological disease, 2022. https://ranzcog.edu.au/wp-content/uploads/Managing-Adnexa-Hysterectomy-Benign-Gynaecological-Disease.pdf (viewed Sept 2025)

92. Royal Australian and New Zealand College of Obstetricians and Gynaecologists. Sacrocolpopexy, 2022. https://ranzcog.edu.au/wp-content/uploads/Sacrocolpopexy.pdf (viewed Sept 2025)

93. National Asthma Council. Australian Asthma Handbook (2nd edition), 2022. https://www.asthmahandbook.org.au/ (viewed Sept 2025)

94. Chung E, Lowy M, Gillman M, Love C, Katz D, Neilsen G. Urological Society of Australia and New Zealand (USANZ) and Australasian Chapter of Sexual Health Medicine (AChSHM). 2022. Royal Australasian College of Physicians (RACP) clinical guidelines on the management of erectile dysfunction. Med J Aust 217:318-324

95. Royal Australian and New Zealand College of Obstetricians and Gynaecologists Caesarean birth on maternal request (C-Obs 39), 2023. https://ranzcog.edu.au/wp-content/uploads/Birth-After-Previous-Caesarean-Section-GDG-ToR.pdf (viewed Sept 2025)

96. Cancer Council. Clinical practice guidelines for the prevention, early detection and management of colorectal cancer, 2023. https://www.cancer.org.au/clinical-guidelines/bowel-cancer/colorectal-cancer (viewed Sept 2025)

97. Society of Obstetric Medicine. SOMANZ Hypertension in Pregnancy Guideline, 2023. https://www.somanz.org/hypertension-in-pregnancy-guideline-2023/ (viewed Sept 2025)

98. Telethon Kids Institute. National healthy skin guideline for the diagnosis, treatment and prevention of skin infections for Aboriginal & Torres Strait Islander Children and Communities in Australia, 2023. https://infectiousdiseases.thekids.org.au/globalassets/media/documents/our-research/healthy-skin-arf/hsg-digital-04-12-2023.pdf (viewed Sept 2025)

99. Caring for Australians and New Zealanders with Kidney Impairment (CARI). CARI Guidelines: Australian and New Zealand living guideline for chronic kidney disease, 2023. https://app.magicapp.org/#/guideline/Lpmozn/section/jz7rbX (viewed Sept 2025)

100. Cancer Council Australia. Clinical practice guidelines for hepatocellular carcinoma surveillance for people at high risk in Australia, 2023. https://app.magicapp.org/#/guideline/7585 (viewed Sept 2025)

101. Centre of Perinatal Excellence (COPE). Mental Health Care in the Perinatal Period, 2023. https://www.cope.org.au/uploads/images/Health-professionals/COPE_2023_Perinatal_Mental_Health_Practice_Guideline.pdf (viewed Sept 2025)

102. Zanker J, Sim M, Anderson K, Balogun S, Brennan-Olsen SL, Dent E, Duque G, Girgis CM, Grossmann M, Hayes A, Henwood T, Hirani V, Inderjeeth C, Iuliano S, Keogh J, Lewis JR, Lynch GS, Pasco JA, Phu S, Reijnierse EM, Russell N, Vlietstra L, Visvanathan R, Walker T, Waters DL, Yu S, Maier AB, Daly RM, Scott D. Consensus guidelines for sarcopenia prevention, diagnosis and management in Australia and New Zealand. 2023 J Cachexia Sarcopenia Muscle 14:142-156

103. Australia and New Zealand Sarcoma Association. Clinical practice guidelines for management of sarcoma, 2023. https://www.sarcoma.org.au/pages/sarcoma-guidelines/preamble (viewed Sept 2025)

104. Living Evidence for Diabetes Consortium. Australian Evidence-Based Clinical Guidelines for Diabetes, 2023. https://app.magicapp.org/#/guideline/E5AbPE (viewed Sept 2025)

105. Royal Australian and New Zealand College of Obstetricians and Gynaecologists. Home births (C-Obs 2), 2023. https://ranzcog.edu.au/wp-content/uploads/Home-Births.pdf (viewed Sept 2025)

106. Commonwealth of Australia as represented by the Department of Health and Aged Care. Australian Guideline for assessing and managing cardiovascular disease risk, 2023. https://www.cvdcheck.org.au/ (viewed Sept 2025)

107. Autism Cooperative Research Centre. National guideline for the assessment and diagnosis of autism in Australia, 2023. https://www.autismcrc.com.au/best-practice/assessment-and-diagnosis (viewed Sept 2025)

108. Royal Australian and New Zealand College of Obstetricians and Gynaecologists C-Obs 32 Responsibility for neonatal resuscitation at birth, 2023. https://ranzcog.edu.au/wp-content/uploads/Neonatal-Resuscitation-at-Birth.pdf (viewed Sept 2025)

109. Royal Australian and New Zealand College of Obstetricians and Gynaecologists. Guidelines for the use of Rh(D) Immunoglobulin (Anti-D) in obstetrics (C-Obs 6), 2023. https://ranzcog.edu.au/wp-content/uploads/Anti-D-Guidelines.pdf (viewed Sept 2025)

110. Royal Australian and New Zealand College of Obstetricians and Gynaecologists Placenta Accreta Spectrum (PAS) (C-Obs 20), 2023. https://ranzcog.edu.au/wp-content/uploads/Placenta-Accreta-Spectrum.pdf (viewed Sept 2025)

111. Royal Australian and New Zealand College of Obstetricians and Gynaecologists Prevention of congenital cytomegalovirus (CMV) infection, 2023. https://ranzcog.edu.au/wp-content/uploads/Prevention-CMV-Infection.pdf (viewed Sept 2025)

112. Royal Australian and New Zealand College of Obstetricians and GynaecologistsC-Obs 37 Delivery of fetus at caesarean birth, 2023. https://ranzcog.edu.au/wp-content/uploads/Delivery-Fetus-Caesarean-Birth.pdf (viewed Sept 2025)

113. Royal Australian and New Zealand College of Obstetricians and Gynaecologists C-Obs 29b Progesterone: Use in the second and third trimester, 2023. https://ranzcog.edu.au/wp-content/uploads/Progesterone-Use-Second-Third-Trimester.pdf (viewed Sept 2025)

114. Royal Australian and New Zealand College of Obstetricians and Gynaecologists. Use of prostaglandins for induction of labour (C-Obs 22), 2023. https://ranzcog.edu.au/wp-content/uploads/Prostaglandins-Induction-of-Labour.pdf (viewed Sept 2025)

115. Royal Australian and New Zealand College of Obstetricians and Gynaecologists. C-Gyn 1 Female Genital Mutilation/Cutting (FGM/C), 2023. https://ranzcog.edu.au/wp-content/uploads/Female-Genital-Mutilation.pdf (viewed Sept 2025)

116. Royal Australian and New Zealand College of Obstetricians and Gynaecologists Pre-pregnancy counselling (C-Obs 3a), 2024. https://ranzcog.edu.au/wp-content/uploads/Pre-Pregnancy-Counselling.pdf (viewed Sept 2025)

117. McDonald CF, Serginson J, AlShareef S, Buchan C, Davies H, Miller BR, et al. Thoracic Society of Australia and New Zealand clinical practice guideline on adult home oxygen therapy. Respirology. 2024; 29(9): 765–784

118. Royal Australian and New Zealand College of Obstetricians and Gynaecologists The use of misoprostol in obstetrics and gynaecology, 2024. https://ranzcog.edu.au/wp-content/uploads/2022/05/The-use-of-misoprostol-in-obstetrics-and-gynaecology.pdf (viewed Sept 2025)

119. Centre of Research Excellence in Stillbirth & Perinatal Society of Australia and New Zealand 2024. Care Around Stillbirth and Neonatal Death Clinical Practice Guideline, available at https://learn.stillbirthcre.org.au/ (viewed Sept 2025)

120. Royal Australian and New Zealand College of Obstetricians and Gynaecologists. Substance use in pregnancy (C-Obs 53), 2024. https://ranzcog.edu.au/wp-content/uploads/Substance-Use-Pregnancy.pdf (viewed Sept 2025)

121. Royal Australian and New Zealand College of Obstetricians and Gynaecologists Care in labour in the absence of pregnancy complications (C-Obs 31), 2024. https://ranzcog.edu.au/wp-content/uploads/Care-Labour-Absence-Pregnancy-Complications.pdf (viewed Sept 2025)

122. Royal Australian and New Zealand College of Obstetricians and Gynaecologists C-Gyn 3 Contraception, 2024. https://ranzcog.edu.au/wp-content/uploads/Contraception-Clinical-Guideline.pdf (viewed Sept 2025)

123. Royal Australian and New Zealand College of Obstetricians and Gynaecologists. Prenatal screening for fetal genetic or structural conditions, 2019. https://ranzcog.edu.au/wp-content/uploads/2022/05/Prenatal-Screening-for-Fetal-Genetic-or-Structural-Condition.pdf (viewed Sept 2025)

124. Royal Australian and New Zealand College of Obstetricians and Gynaecologists. Screening and diagnosis of fetal structural anomalies and chromosome conditions (C-Obs 35), 2024. https://ranzcog.edu.au/wp-content/uploads/Fetal-Anomalies-Clinical-Guideline.pdf (viewed Sept 2025)

125. Royal Australian and New Zealand College of Obstetricians and Gynaecologists. Early pregnancy screening and prevention of preterm preeclampsia and related complications (C-Obs 61), 2024. at https://ranzcog.edu.au/wp-content/uploads/Screening-Prevention-Preterm-PET.pdf (viewed Sept 2025)

126. Lung Foundation Australia. The COPD-X plan: Australian and New Zealand Guidelines for the management of Chronic Obstructive Pulmonary Disease, 2024. https://copdx.org.au/wp-content/uploads/2024/08/COPD-X-V2.75_FINAL.pdf (viewed Sept 2025)

127. National Blood Authority. Patient blood management guideline for adults with critical bleeding, 2024. https://www.blood.gov.au/patient-blood-management-guideline-adults-critical-bleeding (viewed Sept 2025)

128. National Blood Authority. Guideline for the prophylactic use of Rh D immunoglobulin in pregnancy care, 2024. https://www.blood.gov.au/guideline-prophylactic-use-rh-d-immunoglobulin-pregnancy-care (viewed Sept 2025)

129. The BRIDGES guideline development group. Australian Physical Activity Clinical Practice Guideline for people with moderate to severe traumatic brain injury, 2024. https://www.imh.org.au/bridges (viewed Sept 2025)

130. Centre for Research Excellence in Women's Health in Reproductive Life. Australian Evidence-based Guideline for unexplained infertility ADAPTE process from the ESHRE Evidence-based Guideline on unexplained infertility 2024, 2024. https://www.monash.edu/medicine/mchri/infertility/guideline (viewed Sept 2025)

131. Royal Australian and New Zealand College of Obstetricians and Gynaecologists. Pre-pregnancy and pregnancy-related vaccinations (C-Obs 44), 2024. https://ranzcog.edu.au/wp-content/uploads/Pre-Pregnancy-and-Pregnancy-Related-Vaccinations.pdf (viewed Sept 2025)

132. Centre for Research Excellence in Newborn Medicine, Preterm Follow-up Guideline Development Group. Guideline for Growth, Health and Developmental Follow-up for Children Born Very Preterm, 2024. https://www.crenewbornmedicine.org.au/our-news/news/2024/guideline-for-growth-health-and-developmental-follow-up-for-children-born-very-preterm/ (viewed Sept 2025)

133. Royal Australian College of General Practitioners and Healthy Bones Australia. Osteoporosis prevention, diagnosis and management in postmenopausal women and men over 50 years of age, 2024. https://healthybonesaustralia.org.au/wp-content/uploads/2024/03/hba-racgp-guidelines-2024.pdf (viewed Sept 2025)

134. National Aboriginal Community Controlled Health Organisation and The Royal Australian College of General Practitioners. National guide to preventive healthcare for Aboriginal and Torres Strait Islander people: Recommendations. 4th edition, 2024. https://www.racgp.org.au/getattachment/ef5164d0-14ce-40b6-bd2e-7fe38922f7d4/National-Guide-to-preventive-healthcare-for-Aboriginal-and-br-Torres-Strait-Islander-people.aspx (viewed Sept 2025)

135. Royal Australian College of General Practitioners. Supporting smoking cessation: A guide for health professionals, 2024. https://www.racgp.org.au/getattachment/be9bceb1-07bf-4848-8a7f-c0ce48001d2a/Supporting-smoking-cessation-A-guide-for-health-professionals.aspx (viewed Sept 2025)

136. Longhitano A, Roder C, Blackmore T, Campbell A, May M and Athan E Australasian Society of Infectious Diseases updated guidelines for the management of Clostridioides difficile infection in adults and children in Australia and New Zealand. Intern Med J 2025 55: 503-513

137. Living Evidence for Australian Pregnancy and Postnatal Care program. Australian Postnatal Care Guidelines, 2025. https://app.magicapp.org/#/guideline/jW0ZbL (viewed Sept 2025)

138. Living Evidence for Australian Pregnancy and Postnatal Care program. Australian Pregnancy Care Guidelines, 2025. https://app.magicapp.org/#/guideline/10505 (viewed Sept 2025)

139. Royal Australian College of General Practitioners. Guidelines for preventive activities in general practice, 2025. https://www.racgp.org.au/clinical-resources/clinical-guidelines/key-racgp-guidelines/view-all-racgp-guidelines/preventive-activities-in-general-practice/about-the-red-book (viewed Sept 2025)

140. Cancer Council Australia. National Cervical Screening Program Guidelines, 2025. https://www.cancer.org.au/clinical-guidelines/cervical-cancer/cervical-cancer-screening (viewed Sept 2025)

141. Stroke Foundation. Australian and New Zealand Living Clinical Guidelines for Stroke Management. 2025. https://informme.org.au/en/Guidelines/Clinical-Guidelines-for-Stroke-Management (viewed Sept 2025)

142. Royal Australian and New Zealand College of Obstetricians and Gynaecologists. Australian Living Evidence Guideline: Endometriosis, 2025. https://ranzcog.edu.au/wp-content/uploads/Endometriosis-Clinical-Practice-Guideline.pdf (viewed Sept 2025)

143. Royal Australian and New Zealand College of Obstetricians and Gynaecologists. C-Obs 38 Birth after previous caesarean section, 2025. https://ranzcog.edu.au/wp-content/uploads/Birth-After-Previous-Caesarean-Section-GDG-ToR.pdf (viewed Sept 2025)

144. Royal Australian and New Zealand College of Obstetricians and Gynaecologists Clinical guideline for abortion care: An evidence based guideline on abortion care in Australia and Aotearoa New Zealand, 2025. https://ranzcog.edu.au/wp-content/uploads/Clinical-Guideline-Abortion-Care.pdf (viewed Sept 2025)

145. Royal Australian and New Zealand College of Obstetricians and Gynaecologists. Miscarriage, recurrent miscarriage and ectopic pregnancy (C-Gyn 38), 2025. https://ranzcog.edu.au/wp-content/uploads/Miscarriage-Ectopic-Pregnancy.pdf (viewed Sept 2025)

146. Australian Technical Advisory Group of Immunisation (ATAGI). Australian immunisation handbook, 2025. https://immunisationhandbook.health.gov.au/ (viewed Sept 2025)

147. Australia and New Zealand Musculoskeletal (ANZMUSC) Clinical Trials Network, Australian Rheumatology Association (ARA), and Cochrane Musculoskeletal, in conjunction with the NPS MedicineWise consortium.

Australia and New Zealand An Australian Living Guideline for the Pharmacological Management of Inflammatory Arthritis, 2025. https://app.magicapp.org/#/guideline/LqRV3n (viewed Sept 2025)

148. Australian and New Zealand Committee on Resuscitation. Australian and New Zealand Committee on Resuscitation Guidelines, 2025. https://www.anzcor.org/ (viewed Sept 2025)

149. Australia and New Zealand Musculoskeletal (ANZMUSC) Clinical Trials Network, Australian Rheumatology Association (ARA), and Cochrane Musculoskeletal. An Australian living guideline for the management of juvenile idiopathic arthritis, 2025. https://app.magicapp.org/#/guideline/9673 (viewed Sept 2025)

150. Vernon-Roberts A, Chan P, Christensen B, Havrlant R, Giles E, Williams AJ. Pediatric to Adult Transition in Inflammatory Bowel Disease: Consensus Guidelines for Australia and New Zealand. Inflamm Bowel Dis 2025 31:563-578.

Preferred Reporting Items for Systematic reviews and Meta-Analyses extension for Scoping Reviews (PRISMA-ScR) Checklist

The page numbers in this checklist refer to the submitted manuscript, not to the published article or its Supporting Information file.

SECTION	ITEM	PRISMA-ScR CHECKLIST ITEM	REPORTED ON PAGE #
**TITLE**			
Title	1	Identify the report as a scoping review.	Yes
**ABSTRACT**			
Structured summary	2	Provide a structured summary that includes (as applicable): background, objectives, eligibility criteria, sources of evidence, charting methods, results, and conclusions that relate to the review questions and objectives.	1
**INTRODUCTION**			
Rationale	3	Describe the rationale for the review in the context of what is already known. Explain why the review questions/objectives lend themselves to a scoping review approach.	2
Objectives	4	Provide an explicit statement of the questions and objectives being addressed with reference to their key elements (e.g., population or participants, concepts, and context) or other relevant key elements used to conceptualize the review questions and/or objectives.	3
**METHODS**			
Protocol and registration	5	Indicate whether a review protocol exists; state if and where it can be accessed (e.g., a Web address); and if available, provide registration information, including the registration number.	4
Eligibility criteria	6	Specify characteristics of the sources of evidence used as eligibility criteria (e.g., years considered, language, and publication status), and provide a rationale.	4 - 5
Information sources*	7	Describe all information sources in the search (e.g., databases with dates of coverage and contact with authors to identify additional sources), as well as the date the most recent search was executed.	5 - 6
Search	8	Present the full electronic search strategy for at least 1 database, including any limits used, such that it could be repeated.	5
Selection of sources of evidence†	9	State the process for selecting sources of evidence (i.e., screening and eligibility) included in the scoping review.	6
Data charting process‡	10	Describe the methods of charting data from the included sources of evidence (e.g., calibrated forms or forms that have been tested by the team before their use, and whether data charting was done independently or in duplicate) and any processes for obtaining and confirming data from investigators.	6 - 7
Data items	11	List and define all variables for which data were sought and any assumptions and simplifications made.	Supporting Information 2
Critical appraisal of individual sources of evidence§	12	If done, provide a rationale for conducting a critical appraisal of included sources of evidence; describe the methods used and how this information was used in any data synthesis (if appropriate).	N/A
Synthesis of results	13	Describe the methods of handling and summarizing the data that were charted.	7
**RESULTS**			
Selection of sources of evidence	14	Give numbers of sources of evidence screened, assessed for eligibility, and included in the review, with reasons for exclusions at each stage, ideally using a flow diagram.	8 and Supporting Information 3
Characteristics of sources of evidence	15	For each source of evidence, present characteristics for which data were charted and provide the citations.	8 to 10, Supporting Information 4
Critical appraisal within sources of evidence	16	If done, present data on critical appraisal of included sources of evidence (see item 12).	N/A
Results of individual sources of evidence	17	For each included source of evidence, present the relevant data that were charted that relate to the review questions and objectives.	8 to 10
Synthesis of results	18	Summarize and/or present the charting results as they relate to the review questions and objectives.	8 to 10
**DISCUSSION**			
Summary of evidence	19	Summarize the main results (including an overview of concepts, themes, and types of evidence available), link to the review questions and objectives, and consider the relevance to key groups.	11
Limitations	20	Discuss the limitations of the scoping review process.	Click here to enter text.
Conclusions	21	Provide a general interpretation of the results with respect to the review questions and objectives, as well as potential implications and/or next steps.	Click here to enter text.
**FUNDING**			

SECTION	ITEM	PRISMA-ScR CHECKLIST ITEM	REPORTED ON PAGE #
Funding	22	Describe sources of funding for the included sources of evidence, as well as sources of funding for the scoping review. Describe the role of the funders of the scoping review.	Click here to enter text.

JBI = Joanna Briggs Institute; PRISMA-ScR = Preferred Reporting Items for Systematic reviews and Meta-Analyses extension for Scoping Reviews.

* Where *sources of evidence* (see second footnote) are compiled from, such as bibliographic databases, social media platforms, and Web sites.

† A more inclusive/heterogeneous term used to account for the different types of evidence or data sources (e.g., quantitative and/or qualitative research, expert opinion, and policy documents) that may be eligible in a scoping review as opposed to only studies. This is not to be confused with *information sources* (see first footnote).

‡ The frameworks by Arksey and O'Malley (6) and Levac and colleagues (7) and the JBI guidance (4, 5) refer to the process of data extraction in a scoping review as data charting.

§ The process of systematically examining research evidence to assess its validity, results, and relevance before using it to inform a decision. This term is used for items 12 and 19 instead of "risk of bias" (which is more applicable to systematic reviews of interventions) to include and acknowledge the various sources of evidence that may be used in a scoping review (e.g., quantitative and/or qualitative research, expert opinion, and policy document).

From: Tricco AC, Lillie E, Zarin W, O'Brien KK, Colquhoun H, Levac D, et al. PRISMA Extension for Scoping Reviews (PRISMAScR): Checklist and Explanation. Ann Intern Med. 2018;169:467–473.
